# Dataset on controlled production of polyhydroxyalkanoate-based microbead using double emulsion solvent evaporation technique

**DOI:** 10.1016/j.dib.2019.01.023

**Published:** 2019-01-17

**Authors:** Sharumathiy Govindasamy, Ishak Muhammad Syafiq, Al-Ashraf Abdullah Amirul, Roswati Md Amin, Kesaven Bhubalan

**Affiliations:** a School of Marine and Environmental Sciences, UMT, 21030 Kuala Nerus, Terengganu, Malaysia; b Malaysian Institute of Pharmaceuticals and Nutraceuticals, NIBM,11700 Pulau Pinang, Malaysia; c School of Biological Sciences, USM, 11800 Pulau Pinang, Malaysia; d Institute of Marine Biotechnology, UMT, 21030 Kuala Nerus, Terengganu, Malaysia

**Keywords:** *Masillia haematophilla* UMTKB-2, Polyhydroxyalkanoate, Microbeads, Cosmeceutical

## Abstract

A significant source of microplastics is from the usage of microbeads in the market since petrochemical plastic bead is a material used in cosmetic scrubs. A possible way to counteract the problem is by the substitution of synthetic plastic to natural biodegradable polymer. Polyhydroxyalkanoate (PHA) is a general class of thermoplastic microbial polymer and it is the best alternative to some petrochemical plastics due to its biodegradability. Some PHA has earned its way into cosmetic application due to its biocompatibility. This data article reports data on the development of biodegradable microbeads by using the double emulsion solvent evaporation technique. Our data describe the extraction of biopolymer from marine bacteria that was cultivated in shaken flask culture, removal of endotoxins using oxidizing agent, the production of microbeads using a peristaltic pump with a specific flowrate and silicon tubing, and the cytotoxicity of the microbeads.

**Specifications table**

**Table:** 

Subject area	Biology
More specific subject area	Microbiology, Biotechnology, Environmental
Type of data	Scanning electron microscope (SEM) images, tables
How data was acquired	Orbital shaker (Certomat® R & H, Sartorious Sedim Biotech, Germany)
	Spectrophotometer (Genesys 105 UV–vis. (Thermo SCIENTIFIC, USA))
	Centrifuge (Avanti® J-E Centrifuge (Beckman Coulter, USA))
	Freeze drier (Free- Zone Freeze Dry System, LABCONCO, USA)
	Gas chromatography (Shimadzu GC-2010 (Shimadzu, Japan))
	Removal of endotoxin kit (E-TOXATE™ Kits (Sigma Aldrich, USA))
	Peristaltic Pump (Watson Marlow 101U/R, England)
	Digital Homogenizer (Ultra-Turrax T-25, IKA Works, USA)
	Scanning electron microscope (Hitachi Field Emission Scanning
	Electron Microscope – FESEM Model SU8010, Hitachi, Japan)
Data format	Analyzed
Experimental factors	*Bacillus megaterium* UMTKB-1 and *Massilia haematophila* UMTKB-2 culture were incubated for 14 h and 12 h of incubation time, respectively, which is the mid-exponential growth phase
Experimental features	Analysis was done using Watson Marlow 101U/R,
	Hitachi Field Emission Scanning Electron Microscope – FESEM Model SU8010
Data source location	Mengabang Telipot, Kuala Terengganu, Terengganu, Malaysia
Data accessibility	All data is accessible within this article
Related research article	S. Mohamed, A.A. Amirul, A.W.M. Effendy, K. Bhubalan, Characterization and cytotoxicity of polyhydroxyalkanoate microparticles as adjuvant matrix for the immobilization of *Pasteurella multocida* whole-cell vaccine. J. Sustain. Sci. Manag. 12(2) (2017) 89–95.
	A.F.M Yatim, I.M Syafiq, K.H. Huong, A.A. Amirul, A.W.M Effendy, K. Bhubalan, Bioconversion of novel and renewable agro-industry by-products into a biodegradable poly(3-hydroxybutyrate) by marine *Bacillus megaterium* UMTKB-1 strain. Biotechnologia.2 (2017) 141–151.
	J.T Kiun, K. Bhubalan, A.A. Amirul, Novel PHA bioplastic producing bacteria isolated from brackish environment. In the 14th Symposium of Malaysian Society of Applied Biology (2016) 149–155.

**Value of the data**

• This data comprise the methodological data of developing biodegradable microbeads from a novel PHA-producer, *Massilia haematophila* UMTKB-2.
• This data represent the use of automated technique instead of manual pipetting technique to produce controlled bead size with rapid, continuous and high reproducibility.
• This data can be used in future product application for the betterment of our earth by reducing the accumulation of microplastic waste in the ocean.
• This data can be used to replace synthetic plastic microbeads in the market that are manufactured and used in consumer products, such as cosmetic scrubs.
• This data serve as a benchmark as the first report on the production of PHA-based microbeads that could be commercialized as a PHA-based dermal exfoliating scrub.

## 1 Data

**Fig. 1:**
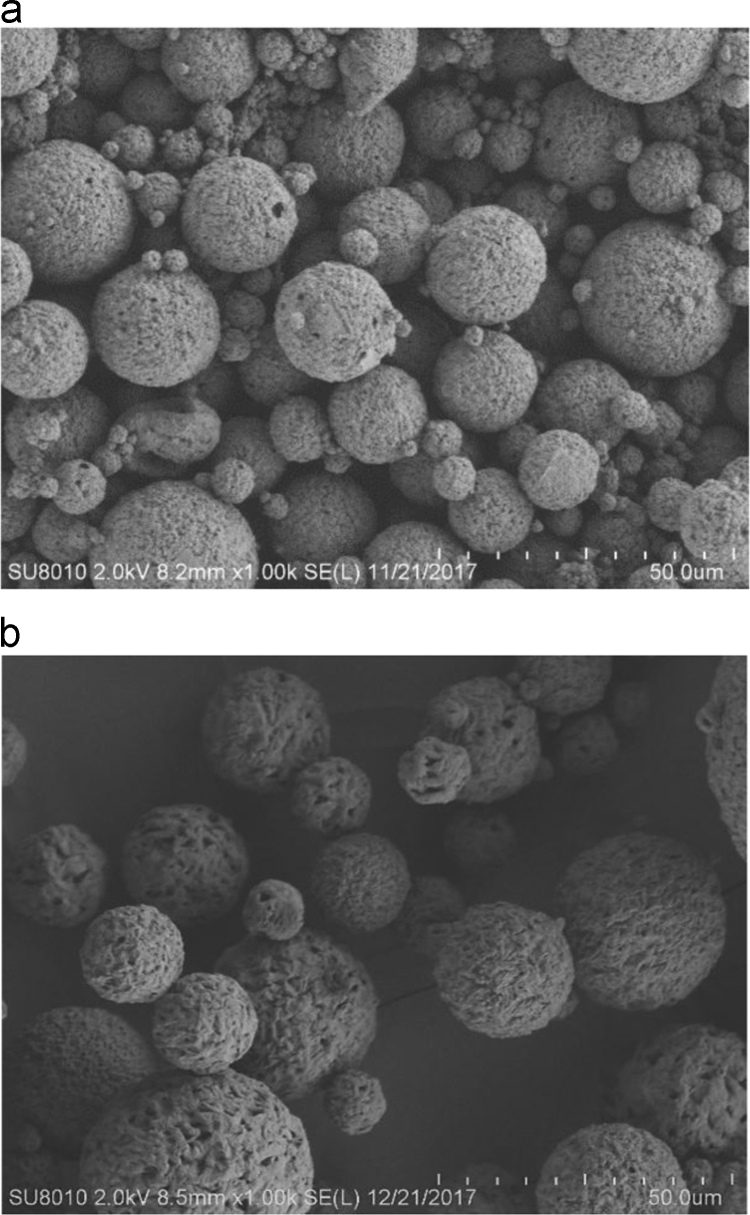
Scanning electron microscopy (SEM) of produced PHA-based microbeads. (a) SEM of homopolymer, P(3HB) microbeads with 1000× magnification. (b) SEM of copolymer, P(3HB-*co*-3HV) microbeads with 1000× magnification.

**Fig. 2:**
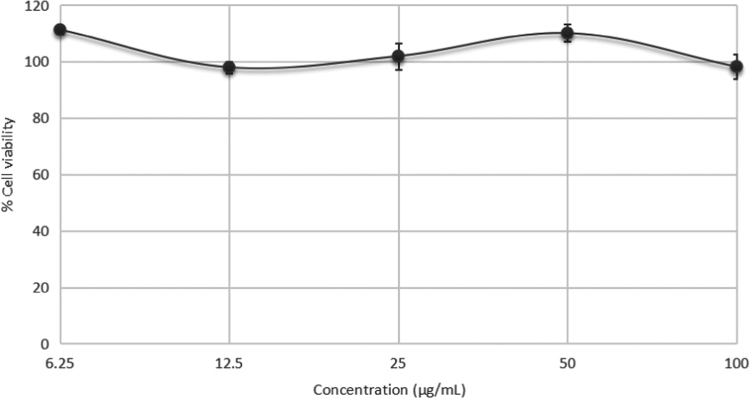
The cell viability of HaCaT cells on the P(3HB)- based microbeads with varying concentrations. Values are mean of four replicates. The error bars represent the standard deviation of the mean (4 S.D).

This data article reports on the methods to derive optimized microbeads from poly(3-hydroxybutyrate) [P(3HB)] homopolymer produced by *Bacillus megaterium* UMTKB-1 and poly(3-hydroxybutyrate-*co*-3-hydroxyvalerate) P(3HB-*co*-3HV) copolymer produced by *Massilia haematophila* UMTKB-2 using the double emulsion solvent evaporation technique. Fig. 1 shows the scanning electron microscope (SEM) images of the produced PHA-based microbeads. Table 1 represents the SEM images showing various sizes of the microbeads. The size of commercially available synthetic microbead ranges from 8 to 56 µm [1] while human skin pore size ranges from 250 to 500 µm [2]. The PHA-based microbead produced in this data article ranged from 10.1 to 140 µm with an average diameter of 38.44 µm, which is more compatible with human skin pore size than the microbead sizes reported by Mohamed and co-workers that ranged from 0.3 to 0.6 µm [3], and those of Murueva and co-workers that ranged from 0.7 to 2.6 µm [4]. The endotoxin level of polymer recovered by the chloroform extraction from the Gram-negative *Massilia haematophila* UMTKB-2 was recorded at 30.72 EU/g. A drastic decrease of endotoxin level to 0.24 EU/g was observed after pyrogen removal using oxidizing agent. *In vitro* cell culture was carried out using human keratinocyte cells (HaCaT) on the P(3HB) microbeads to evaluate the cytotoxicity. There were no IC_50_ value recorded on the HaCaT cells as shown in Fig. 2.

**Table 1:** The scanning electron microscopy (SEM) images showing various sizes of microbeads.

## 2 Experimental design, materials and methods

The strains used in this experiment were *Massilia haematophila* UMTKB-2, isolated from brackish water in Mengabang Telipot, Kuala Terengganu, Terengganu, Malaysia [5] and *Bacillus megaterium* UMTKB-1, isolated from the tissue sample of marine sponges collected from Pulau Langkawi, Kedah, Malaysia [6]. *Bacillus megaterium* UMTKB-1 was employed to synthesize homopolymer P(3HB), while *Massilia haematophila* UMTKB-2 was employed to synthesize copolymer P(3HB-*co*-3HV). The bacterial strains were streaked on nutrient rich (NR) agar routinely. *Bacillus megaterium* UMTKB-1 and *Massilia haematophila* UMTKB-2 were first cultured into a sterile NR medium using shaken flask cultivation method for 14 and 12 h respectively to activate the cells at 200 rotations per minute (rpm) until the mid-exponential growth phase, after which the growth phase was determined by measuring the optical density of the bacterial culture at 600 nm. The biosynthesis, harvesting and recovery of P(3HB) was carried out according to Yatim and co-workers using sweet water [6], whereas the biosynthesis, harvesting and recovery of P(3HB-*co*-3HV) was performed according to Kiun and co-workers [5]. Inactivation and removal of endotoxins from the polymers were performed using hydrogen peroxide, after which the endotoxin levels were tested using E-TOXATE™ Kits (Sigma Aldrich) [7].

PHA microbeads were prepared by the double emulsion solvent evaporation technique with some modifications to obtain larger particles using automated technique [3], [4]. Approximately 0.4 g PHA was dissolved in 10 mL of dichloromethane. The dissolved solution prepared for emulsification was continuously extruded at a flow rate of 1.62 mL/min, with a 3.2 mm silicone tubing using a Watson Marlow 101U/R peristaltic pump into 0.5% (w/v) of polyvinyl alcohol (PVA). It was then homogenized at 10,000 rpm using Ultra-Turrax T-25 digital homogenizer until the solvent had completely evaporated. All emulsions were continuously mixed mechanically for 24 h, until the remaining solvent had completely evaporated. The microbeads were extracted by suction filtration using 0.2 µm nylon-66 filter paper, washed three times with distilled water, and dried overnight for scanning electron microscope analysis. The size range of the microbeads was analyzed by using 20 homopolymer and 20 copolymer beads respectively. Human keratinocyte cell culture (HaCaT) was prepared by culturing in DMEM (Modified Eagle Medium), incubated with sodium pyruvate, penicillin-streptomycin and fetal bovine serum (FBS), then detached by trypsinization according to Chee and co-workers [8]. Cells were seeded at 1 ×105 cells/mL in each well and were incubated at 37 °C in 5% CO_2_ for 24 h. The sample (P3HB-based microbeads) was diluted to 100 µg/mL. The diluted sample was transferred to 96-well flat bottom culture plate and left for incubation at 37 °C in 5% CO_2_ for 24 h. The cell viability was assayed with MTT (3-(4,5-dimethylthiazol-2-yl)-2,5-diphenyltetrazolium bromide) and the absorbance was read after 2 h at 570 nm.

## References

[1] Napper I.E., Bakir A., Rowland S.J., Thompson R.C. (2015). Characterisation, quantity and sorptive properties of microplastics extracted from cosmetics. Mar. Pollut. Bull..

[2] Flament F., Francois G., Qiu H., Ye C., Hanaya T., Batisse D., Cointereau-Chardon S., Seixas M.D.G., Belo S.E.D., Bazin R. (2015). Facial skin pores: a multiethnic study. Clin. Cosmet. Investig. Dermatol..

[3] Mohamed S., Amirul A.A., Effendy A.W.M., Bhubalan K. (2017). Characterization and cytotoxicity of polyhydroxyalkanoate microparticles as adjuvant matrix for the immobilization of Pasteurella multocida whole-cell vaccine. J. Sustain. Sci. Manag..

[4] Murueva A., Shishatskaya E., Kuzmina A., Volova T., Sinskey A. (2013). Microparticles prepared from biodegradable polyhydroxyalkanoates as matrix for encapsulation of cytostatic drug. J. Mater. Sci. Mater. Med..

[5] J.T. Kiun, K. Bhubalan, A.A. Amirul, Novel PHA bioplastic producing bacteria isolated from brackish environment, In: Proceedings of the 14th Symposium of Malaysian Society of Applied Biology, 2016, pp. 149–155.

[6] Yatim A.F.M., Syafiq I.M., Huong K.H., Amirul A.A., Effendy A.W.M., Bhubalan K. (2017). Bioconversion of novel and renewable agro-industry by-products into a biodegradable poly(3-hydroxybutyrate) by marine Bacillus megaterium UMTKB-1 strain. Biotechnologia.

[7] Rao U., Kumar R., Balaji S., Sehgal P. (2010). A novel biocompatible poly (3-hydroxy-co-4-hydroxybutyrate) blend as a potential biomaterial for tissue engineering. J. Bioact. Compat. Polym..

[8] Chee J.W., Amirul A.A., Tengku Muhammad T.S., Majid M.I.A., Mansor S.M. (2008). The influence of copolymer ratio and drug loading level on the biocompatibility of (P3HB-co-4HB) synthetized by Cupriavidus sp. (USMAA2-4). Biochem. Eng. J..

